# Driving as a Travel Option for Older Adults: Findings From the Irish Longitudinal Study on Aging

**DOI:** 10.3389/fpsyg.2019.01329

**Published:** 2019-06-06

**Authors:** Michael Gormley, Desmond O’Neill

**Affiliations:** ^1^School of Psychology, Trinity College Dublin, University of Dublin, Dublin, Ireland; ^2^Centre for Ageing, Neuroscience and the Humanities, Trinity College Dublin, University of Dublin, Dublin, Ireland

**Keywords:** older drivers, driving cessation, driving status, travel options, driving frequency

## Abstract

The role of transport in the health and wellbeing of older people is increasingly recognized: driving is the main form of personal transportation across the adult life-span. Patterns of changed mobility and driving cessation are an important focus of research. We investigated cross-sectional changes in driving as the main form of transportation and the frequency of such driving. The impact of Gender and Marital Status on Driver Status was also examined along with the reasons cited for ceasing driving. The impact that Driver Status had on Quality of Life and Loneliness was also assessed. Questionnaire based data from the Irish longitudinal study on aging (TILDA), a stratified clustered sample of 8163 individuals representative of the community dwelling population aged 50 years and over between 2009 and 2011 were examined. Driving oneself was identified by 76.1% as their most frequently used form of transport. Only for 80+ participants in Rural and Urban non-Dublin was it the second most popular option, being replaced by Being driven by someone else. Less women identified Driving oneself as their most frequently used option and they experienced an almost linear decline in uptake with Age. The uptake reported by men remained high up to 69 and only after this point did it begin to decline. A greater proportion of men were Current drivers with a similar pattern being shown by women in relation to Never drivers. Irrespective of Gender, married participants were more likely to drive. A greater proportion of women cited a reason other than health for giving up driving. Three reasons for giving up were impacted by Age category of which Physical incapacity was not one. Driving status impacted positively on Quality of Life and Loneliness. The results are discussed in light of the advantages to society of older drivers continuing to drive.

## Introduction

One of the most striking trends in the fields of transport, health and aging has been a shift from a previous misplaced emphasis on the safety of older drivers to a realization that a lack of transport access and equity is likely to be a significant threat to well-being and health in later life (O’Neill, 2015). We now know that older drivers are not only a safe group of drivers, but also that their crash rates and fatalities continue to decline (Cicchino and McCartt, 2014) even despite the higher levels of fragility that increases the risk of fatality compared to younger people for a crash of a given severity.

An early indicator of the challenge to transport access and equity was the finding by Foley et al. (2002) that older men and women aged from 70 to 74 could expect not to be driving and would be dependent on alternative transportation for the last 7 and 10 years of their life, respectively. The impacts of driving cessation are well recognized in terms of depression, premature admission to nursing home and mortality (Chihuri et al., 2016). In addition, the association of better health and well-being is recognized with increased life-space mobility, a standard measure of mobility and transport mobility (Portegijs et al., 2016): this is of significance as not driving a car is associated with restricted life-space mobility for older people (Tsuji et al., 2018). The use of the personal car as driver is a key element, not only because it is the primary mode of personal vehicular transport, even in countries with well-developed public transport, but its use as a passenger as opposed to a driver is associated with life-space restriction.

Therefore, there is a strong imperative to understand and interrogate the changes and transitions in late-life transport mobility so as to plan and develop policies and strategies which facilitate the least possible restriction on life-space mobility. One United States cross-sectional study confirmed the gendered decline in daily trips and personal driving with advancing age, but showed that while older women had less daily drips as a driver, they were more likely to travel as a passenger and underwent longer journeys than older men (Shen et al., 2017). There are still many knowledge gaps relating to the transition from driving to non-driving, with factors including health, confidence, comfort and input from family and peers (Dickerson et al., 2017). Among the unresolved issues are the use of multi-modality in transport, as well as how and by whom alternative modes of transport are provided, as well as the impact of higher levels of public transport options in jurisdictions outside the United States.

Longitudinal studies on aging represent an important source of data for exploring driving and transport mobility, although to date many such studies have not included significant data on driving (Bartley and O’Neill, 2010). The Irish Longitudinal Study on Aging (TILDA) offered the opportunity to investigate the travel choices of older Irish adults within the constraints of the questions that were posed by the original survey (Kearney et al., 2011). Issues addressed here in this exploratory analysis cover five main issues. Firstly, which modes of transport were most commonly used and whether location and age impacted on these choices. Secondly, since driving oneself is universally the most common travel choice for older people it is important to determine just how dominant it is in Ireland and whether reliance on it is affected by age and gender. Thirdly, to look at the proportions who have ceased driving or never drove in the first place, and establish the extent to which these are affected by gender. Fourthly, to investigate the reasons cited for giving up and the impact that gender might have on these. And finally, since the ability to drive impacts positively on quality of life and felt loneliness, how being able to drive oneself impacts on these.

## Materials and Methods

TILDA was designed to collect data on a comprehensive set of variables relating to health, economic and social circumstances from participants aged 50 and over. Data collection occurred every 2 years and the first trench were collected between 2009 and 2011, and this trench only was selected for analysis here since it contained the largest sample of participants, with each participant being sampled only once during this time period. These data were collected using Computer-Aided Personal Interview (CAPI), a self-complete questionnaire and physical assessment. Only a very small subset of these data are relevant to the analysis conducted here. The data relating to travel were collected using CAPI. Fifteen questions were posed relating to travel choices, behavior and experiences. Quality of life was measured using the Quality of Life Scale (CASP-19) which measures four domains (control, autonomy, pleasure and self-realization) with Cronbach’s alphas between 0.6 and 0.8 (Hyde et al., 2003) and the data were collected during the CAPI session. Loneliness was measured using the University of California, Los Angeles Loneliness Scale which is a global bipolar factor with Cronbach’s alpha ranging from 0.89 to 0.94 (Russell, 1996). These data were collected during the self-complete questionnaire session.

### Participants

The target population for this research was anyone in the Republic of Ireland, aged over 49, who lived in the community. Postal addresses in Ireland were stratified by socioeconomic status and geographical location, assigned to clusters and then a sample of these clusters were selected. Subsequently 25600 addresses were identified and visited by an interviewer of which 22321 were occupied. Of these, 9818 had a person over 49 and successful interviews were conducted in 6279, leading to a response rate of 62% and a final sample of 8163 (Kenny et al., 2010). As can be seen from Table 1, the sample had slightly more females (54.2%) and an average age of 63.68 (9.16). The three levels of highest education were fairly evenly distributed with Secondary being the most common at 40%. The majority of the sample were married (69%) and rural location was the most common domicile location. In terms of self-rated physical health, 76.8% rated themselves as good or better.

**Table 1:** Sample characteristics.

		Women	Men	Overall
n		4423 (54.2%)	3740 (45.8%)	8163 (100%)
Mean age		63.41 (9.22)	63.68 (9.08)	63.68 (9.16)
HLoE^∗^ – Primary		1247 (28.2%)	1245 (33.3%)	2492 (30.5%)
HLoE – Secondary		1807 (40.8%)	1454 (38.9%)	3261 (40%)
HLoE – Tertiary		1359 (30.8%)	1038 (27.8%)	2397 (29.4%)
Married/living together		2847 (64.4%)	2784 (74.4%)	5631 (69%)
Never married		346 (7.8%)	444 (11.9%)	790 (9.7%)
Separated/divorced		342 (7.7%)	209 (5.6%)	551 (6.7%)
Widowed		888 (20.1%)	303 (8.1%)	1191 (14.6%)
Live in Dublin – city/county		1074 (24.3%)	858 (22.9%)	1932 (23.7%)
Live in town/city – not Dublin		1249 (28.2%)	1059 (28.3%)	2308 (28.3%)
Live rurally – not Dublin		2095 (47.4%)	1816 (48.6%)	3911 (47.9%)
Self-rated physical health	Excellent	729 (16.5%)	543 (14.5%)	1272 (15.6%)
	Very good	1245 (28.1%)	1087 (29.1%)	2332 (28.6%)
	Good	1432 (32.4%)	1227 (32.8%)	2659 (32.6%)
	Fair	788 (17.8%)	694 (18.6%)	1482 (18.2%)
	Poor	229 (5.2%)	188 (5%)	417 (5.1%)

*^∗^HLoE, Highest level of education.*

## Results

### Transport Options Most Frequently Used

**FIGURE 1:**
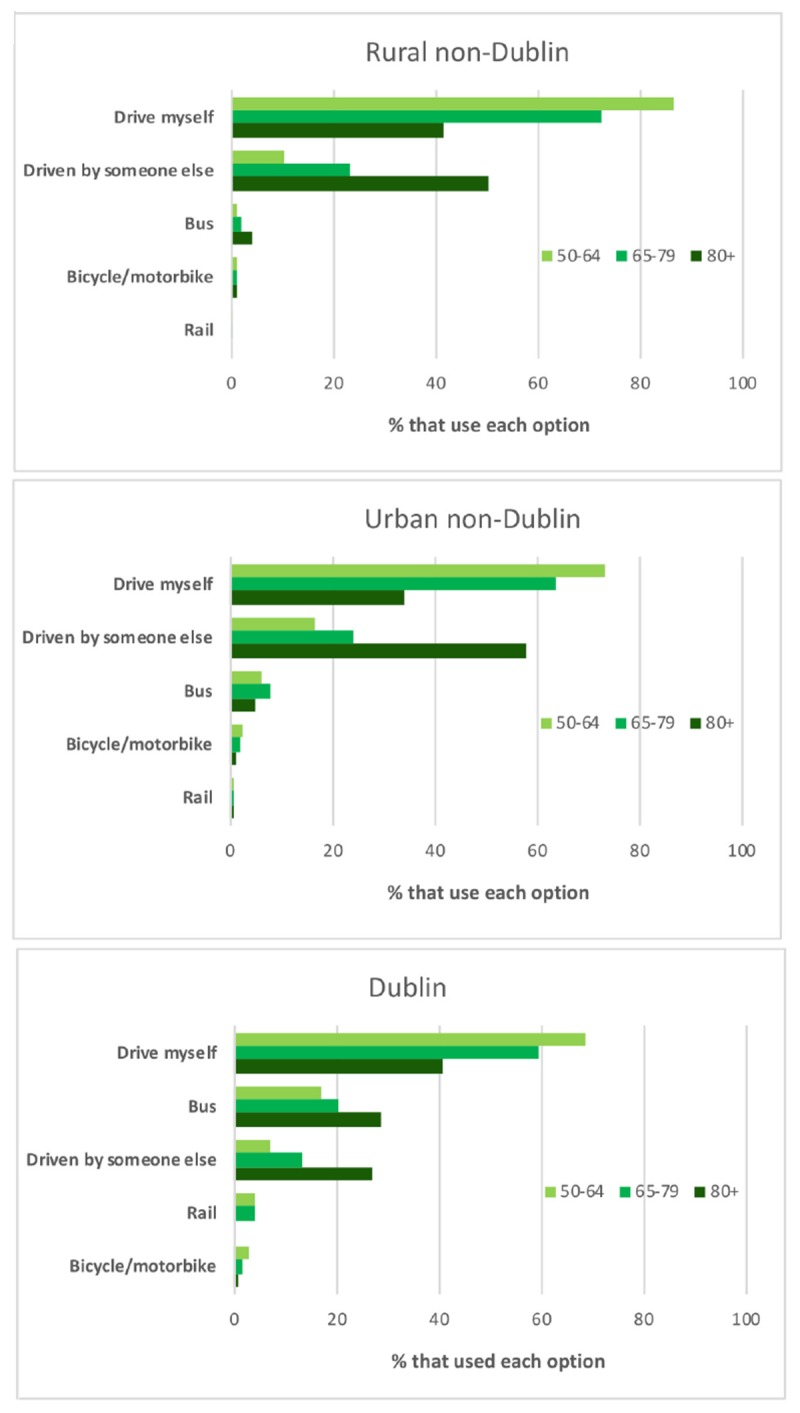
Modes of transport most commonly used across Location and Age^2^.

Participants were asked a single question relating to which of 12^1^ categories of transport options they used most often. For simplicity, these 12 categories were collapsed across the five presented in Figure 1. Driving oneself was by far the most prevalent with 76.1% compared to the next most popular of Been driven my someone else at 17.5%. Slightly different patterns emerged when the data were broken down across Age and Location as depicted in Figure 1. The dominance of Driving oneself remained such that even for the 65–79 cohort the smallest advantage it has over the second most common option was 49% in Dublin where the bus was most popular after Driving oneself. Only in the case of the 80+ participants in Rural and Urban non-Dublin where Driving oneself dropped to 41.7 and 33.9% was been Driven by someone else more popular with 50.5 and 57.9%, respectively. Also of note is the fact that in Dublin the least favorite option was Bicycle/motorbike replacing Rail which occupied this status within the other two regions.

### Driving Oneself

**FIGURE 2:**
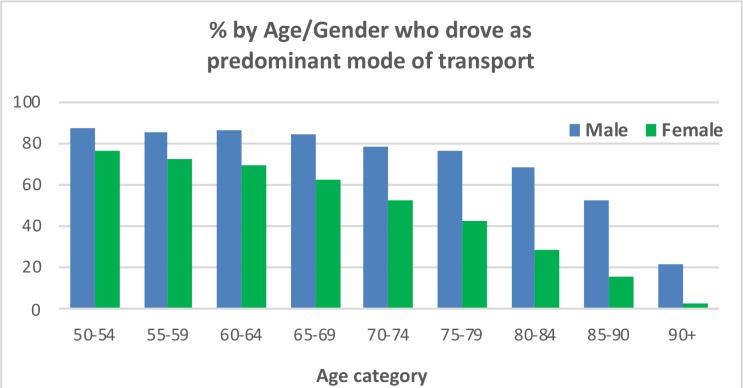
Percentage of participants across Age and Gender who drove themselves as their predominant mode of transport.

Due to the pre-eminence of Driving oneself, it is important to look at these data alone and determine how they break down across Age and Gender, as presented in Figure 2. For males, there is a non-linear decline in Driving oneself as the dominant mode in that prevalence remains above 80% until the 65–69 category and then begins to gradually decline such that at 80–84 it becomes 68.4% and only drops below 50% to 21.4% for the oldest category. For women, however, Age brings about an almost linear decline in the dominance of Driving oneself, starting from 76.9% among 50–54 year-olds, dropping below 50% for the 75–79 (42.7%) and reducing to 29% for the 80–84 s. Thereafter the decline became more pronounced reducing to only 2.9% in the 90+.

**FIGURE 3:**
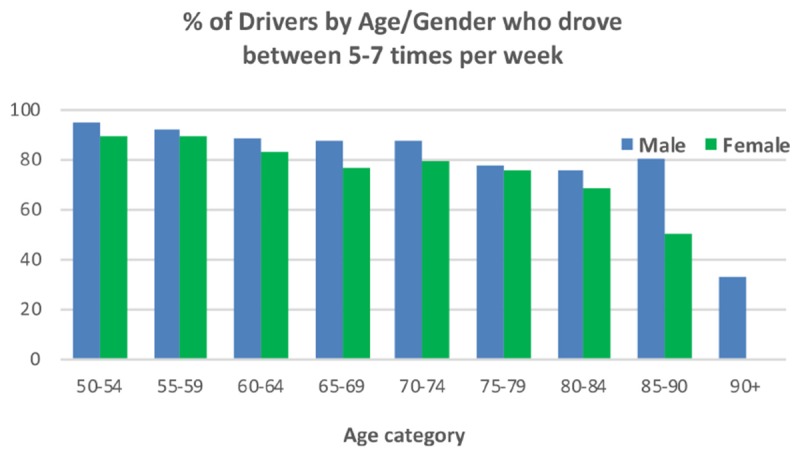
Percentage of Drivers across Age and Gender who drove between 5 and 7 times per week.

In addition to the categorical dominance of Driving oneself, it is important to look at how frequently such driving is engaged in. Of the 5840 identified drivers (classified as such if they had driven at least twice in the last 12 months), 86.9% drove between 5 and 7 days per week. Figure 3 shows the percentage of drivers in each Age/Gender cohort that drove with this frequency. It indicates there was little or no impact of Gender on the slow reduction in this level of driving with 50–54 males starting at 95.1% and dropping to 75.6 for the 80–84 cohort. The equivalent drop in women was of a similar magnitude and rate going from 90.1 to 68.7%. Thereafter the data appear somewhat anomalous (male level increases to 80.9% and female 90+ goes to zero) which is likely to be a by-product of the small numbers remaining driving in these cohorts.

### Driving Status

Current drivers (within the last 12 months drove themselves more than twice) made up 76.1% of the sample, with Ceased drivers (self-identified as having driven in the past, but not more than twice within the last 12 months) and Never drivers making up 7.3 and 16.6%, respectively. A chi-square test of independence indicated that there was a significant relationship between Gender and Driving Status, χ^2^(2, *n* = 8163) = 535.98, *p* < 0.001. Table 2 below shows the biggest deviations from the expected values came from the fact that a larger proportion of women never learned to drive, 25.2% (SR = 14.1) compared to 6.4% (SR = −15.3) for men. In addition, a greater proportion of men were Current drivers, 86.7% (SR = 7.4) as opposed to 67.1% (SR = −6.8) for women. Although the proportions of Males (7%) and Females (7.6%) who gave up driving are remarkedly similar it is nevertheless informative to investigate potential differences between these two groups in terms of reasons for giving up. Note since there were no data available relating to age at which they gave up driving, comparing the age of Males and Females who had given up is uninformative.

**Table 2:** Crosstabulation of gender by driving status.

		Current drivers	Ceased driving	Never drivers	Total
Male	Count	3241	260	239	3740
	Gender%	86.7%	7.0%	6.4%	100%
	*SR*	7.4	−0.8	−15.3	
Female	Count	2970	337	1116	4423
	Gender%	67.1%	7.6%	25.2%	100%
	*SR*	−6.8	0.8	14.1	
Total	Count	6211	597	1355	8163
	Gender%	76.1%	7.3%	16.6%	100%

*SR, standardized residual.*

### Impact of Marital Status on Driver Status

The fact that Gender impacted on Driver status could be explained by the fact that in marriage, males may be more likely to do the driving (Wilkins et al., 1999). Therefore, it is important to consider how Marital Status impacts on Driver Status and since Driver Status is different across males and females it is important to conduct this analysis separately for both genders. As can be seen from Table 3, Gender had little impact on how Driver Status was distributed across Married participants. For Married Males there was a higher than expected number of Drivers (SR = 2.9) and a lower than expected number of Ceased (SR = −4.9) and Never Drivers (SR = −5.6). For Married Females the pattern was very similar although the proportion of Drivers was higher For ease of interpretation the number of Age categories was reduced to three with the first two spanning 15 years. (SR = 3.9) and the proportion of Ceased Drivers was not as low (SR = −2.2). For those who were Never Married, Gender did impact on Driver Status. For Males, the proportion of Drivers was lower than expected (SR = −5.2) and the proportions of Ceased (SR = 6.5) and Never Drivers (SR = 12.5) were higher than expected with the latter representing the most extreme value in the table. A very different pattern emerged for Females in that the distribution of Driver Status was remarkably close to expectation across all 3 levels of Driver Status with −0.3 (for Ceased Driving) being the most extreme value. Being separated or divorced had little impact on the distribution of Driver Status across both Males and Females. The only standardized residual to exceed a magnitude of 2 came from Males who ceased driving, having a value of 3. Being widowed seemed to negatively impact on driving with negative SRs for Current Drivers, −7.3 in the case of Females, and positive SRs for having ceased or never driven with SRs being generally more extreme for Females.

**Table 3:** Crosstabulation of martial status by driving status for both genders separately.

Gender	Driver Status	χ^2^ stats	Marital status			
			Married	Never married	Separated/Divorced	Widowed
Male	Current driver	Count	2556	282	169	234
		%	78.9%	8.7%	5.2%	7.2%
		*SR*	2.9	−5.2	−0.9	−1.8
	Ceased driving	Count	125	67	26	42
		%	48.1%	25.8%	10.0%	16.2%
		*SR*	−4.9	6.5	3.0	4.6
	Never driver	Count	103	95	14	27
		%	43.1%	39.7%	5.9%	11.3%
		*SR*	−5.6	12.5	0.2	1.7
Female	Current driver	Count	2082	234	237	417
		%	70.1%	7.9%	8.0%	14.0%
		*SR*	3.9	0.1	0.5	−7.3
	Ceased driving	Count	185	25	28	99
		%	54.9%	7.4%	8.3%	29.4%
		*SR*	−2.2	−0.3	0.4	3.8
	Never driver	Count	580	87	77	372
		%	52.0%	7.8%	6.9%	33.3%
		*SR*	−5.2	0.0	−1.0	9.9

*SR, standardized residual.*

### Reasons for Ceasing Driving

**FIGURE 4:**
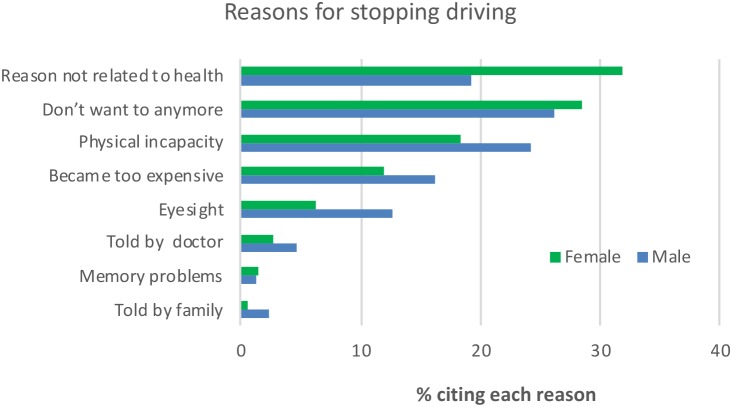
Percentage of participants by Gender who identified each reason for them giving up driving.

Overall the top three cited reasons for giving up driving were Don’t want to anymore (27.5%), Reason not related to health (26.3%) and Physical incapacity (20.9%). Figure 4 presents the percentage of drivers by Gender who agreed that their stopping driving was related to the specified option, with participants being allowed to select as many options as they deemed relevant. Women deviated from the overall trend in that Reason not related to health was most widely cited at 31.8% and this proportion along with the corresponding 19.2% for men produced a significant impact of Gender on identifying this as a reason, χ^2^(1, *n* = 597) = 11.87, *p* < 0.001. The only other reason to produce a significant difference was Problems with eyesight with 12.7% of men citing it as opposed to 6.2% for women, χ^2^(1, *n* = 597) = 7.45, *p* = 0.006^∗^^2^.

In light of the impact of age on health it is important to look at its impact on Reasons for ceasing driving. The same analysis as for Gender was conducted with Age (50–64, 65–79, 80+) replacing Gender and the results are presented in Table 4.

**Table 4:** Key results from chi-square goodness of fit for age category by reason for ceasing.

Reason for ceasing	Overall% citing reason	χ^2^ prob	χ^2^ stats	50–64	65–79	80^+^
Don’t want to anymore	27.5	0.0688	Count	63	66	35
			%	38.4	40.2	21.4
			*SR*	−0.2	−0.3	0.7
Not related to health	26.3	0.256	Count	65	69	23
			%	41.4	43.9	14.6
			*SR*	0.4	0.4	−1.3
Physical incapacity	20.9	0.728	Count	49	55	21
			%	39.2	44	16.8
			*SR*	0.0	0.4	−0.6
Became too expensive	13.7	0.002	Count	43	34	5
			%	52.4	41.5	6.1
			*SR*	1.9	0.0	−2.7
Visual impairment	9	<0.001	Count	10	22	22
			%	18.5	40.7	40.7
			*SR*	−2.4	−0.1	3.6
Told by doctor	3.5	0.798	Count	7	9	5
			%	33.3	42.9	23.8
			*SR*	−0.4	0.1	0.5
Told by family	1.3	0.006	Count	2	1	5
			%	25	12.5	62.5
			*SR*	−0.6	−1.3	2.8
Memory problems	1.3	0.794	Count	4	3	1
			%	50	37.5	12.5
			*SR*	0.5	−0.2	−0.4

*All 3 significant results remain significant at the Bonferroni adjusted alpha level of 0.00625.*

Only three reasons were impacted by Age category and perhaps surprisingly Physical incapacity was not one of them. Becoming too expensive was more frequently cited than expected by the youngest (50–64) group (SR = 1.9) and less frequently by the oldest (80+, SR = −2.7). Visual impairment increased with age with the youngest group citing it less than expected (SR = −2.4) and the oldest group citing it more (SR = 3.6). Being Told by family increased across the age categories and the greatest deviation from the expected frequency came for the oldest group where the standardized residual was 2.8. It is worth noting no such pattern emerged with being Told by doctor where the result was clearly non-significant.

To determine the impact of Driver Status on Quality of Life a three-way factorial ANOVA was conducted with Gender and Age^3^ also included as between groups variables. All three variables produced significant results as presented in Table 5, while all two-way and three-way interactions were non-significant. However, the significant results should be interpreted in light of the effect sizes produced. Partial eta squared for both Gender and Age were almost negligible whereas there was a small impact of Driving Status of 0.024. Tukey *post hoc* tests indicated that the Drivers’ mean score of 44.92 (7.34) was significantly higher than that for Ceased drivers (41.99, SD = 8.77) and Never drivers (41.77, *SD* = 8.48), while the difference between Ceased Drivers and Never drivers was not significant.

**Table 5:** Main effect results for the three between groups variables on quality of life.

Variable	*F*	*df*	*p*	$\eta_{p}^{2}$
Gender	18.523	1,5861	<0.001	0.003
Age	11.479	2,5861	<0.001	0.004
Driving status	71.606	2,5861	<0.001	0.024

A similar three-way ANOVA was also conducted on Loneliness. Again all three variables produced significant results, but as presented in Table 6, only Driver Status produced an effect size of any magnitude (0.015) with Drivers being less lonely that the other two groups. In addition there was an interaction between Gender and Driver Status such that for Ceased drivers the differences between males (2.8, *SD* = 2.57) and females (2.37, *SD* = 2.46) tended toward significance (*p* = 0.072) suggesting men were more lonely.

**Table 6:** Significant results for the three between groups variables on loneliness.

Variable	*F*	*df*	*p*	$\eta_{p}^{2}$
Gender	6.33	1,6659	=0.012	0.001
Age	4.243	2, 6659	=0.014	0.001
Driving status	51.623	2, 6659	<0.001	0.015
Gender ^∗^ Driv Stat	6.043	2, 6659	=0.002	0.002

## Discussion

The pre-eminence of the car as the most often used mode of transport is confirmed by the fact that when Driving oneself and being Driven by someone else are combined, 93.6% of the sample choose either of these two options. Driving oneself at 76.1% for the sample overall was the most popular travel option and even within the 65–79 is was clearly more prevalent mirroring results from other jurisdictions (e.g., Hjorthol et al., 2010). Only among the 80+ cohort within Rural and Urban non-Dublin was its dominance replaced by being Driven by someone else. These data clearly demonstrate that older Irish adults rely upon driving oneself as their most popular form of transport and given the benefits of this it is important to ensure that access to driving is not unnecessarily hindered, e.g., by unwarranted medical screening of older drivers (O’Neill, 2012), while facilitating access to alternative transportation using private or hired cars such as through the creative ITNAmerica system (Bird et al., 2017). With the increase in the level of automation within cars, driving may increasingly become easier and safer making it arguably even more attractive to older people (Harper et al., 2016). Given the advantages of maintaining driving status for older people it is imperative that manufacturers of such cars take into consideration the characteristics and needs of the older driver to ensure that this cohort is not excluded from such advances in technology. In Dublin, the bus was the second most common travel option, so obviously where there is access to appropriate public transport older people will avail of it, but it is important to acknowledge that public transport may not be accessible or adequate for social inclusion once the older person is no longer driving (Hine and Mitchell, 2017). In addition, there are safety concerns for older people in terms of non-collision injuries (O’Neill, 2016).

The usual finding of men driving more than women (Li et al., 2012) is confirmed here in that across all age cohorts men drove more than women (a higher percentage identified it as their most frequently used mode of transport). In addition, these data show how Gender impacts on the decline of the prevalence of driving with Age. For women there is an almost linear decline whereas for men prevalence remains almost static up to 69 years of age and an obvious decline is only seen after 79. The reason for this greater rate of decline needs further investigation. Some diseases vary in prevalence between men and women, e.g., in the TILDA study, hypertension, angina, and stroke are more common in men while osteoporosis, arthritis and high cholesterol are more common in women. Although women report far greater “fear of falling,” no difference in falls prevalence is observed between older men and women (Barrett et al., 2011). Other factors may also play a role, such as pain and urinary incontinence, both of which are more common in women in TILDA. It is also possible the reason may be psychological in nature relating to something like confidence and as yet undetected by the research literature. When looking at the frequency of driving, the previous advantage enjoyed by men is considerably reduced suggesting that when women do choose to drive, they do so just as frequently as men. Only when it comes to the two oldest age categories is there an obvious greater frequency of driving among men.

Following on from the prevalence of driving as the most popular travel option, it is perhaps not surprising to find that Gender and Driver Status are related to each other. When these variables were cross tabulated the biggest deviation from the expected values came from the higher proportion of women who never learned to drive as opposed to the lower proportion of men. Presumably when this cohort was entering early adulthood it was more important for men to learn to drive. Correspondingly a higher than expected proportion of men and a lower than expected proportion of women were Current drivers. Interestingly the proportions of each gender who ceased driving were remarkably similar. Irrespective of Gender, Married participants were more likely to be Drivers with Females being slightly more so, less likely to have ceased driving, with Men slightly less so and less likely to have never driven. It is as if the ability to drive confers an advantage to becoming married, or being married requires development of driving skills and requires this skill to be maintained irrespective of Gender. Arguably, having children may require this skill (Scheiner and Holz-Rau, 2017) and subsequently having grandchildren may necessitate its maintenance into older age. This interpretation is somewhat supported when those who never married are considered. Males who never married are less likely to be a Current Driver and more likely to have ceased driving or to have been a never driver with the latter category providing the most extreme value of this analysis (*SR* = −12.5). This supports the idea that marriage is selective for driving and without the consequences of marriage, never married males are more likely to give up driving. However, this argument is somewhat negated by the fact that never having been married would appear to have no influence on the distribution of Driver Status across Females. This leads to the interpretation that Driver Status is selective for Marriage in Males only, or at least in the era when this sample was getting married, but once married it is selective for developing and maintaining driving skills irrespective of Gender. Being widowed has a negative impact on driving, consistent with other studies (Isherwood et al., 2017): possible interpretations include less practice and attachment to driving by female widows, poor health, increased age and a more realistic sense of driving abilities (Hjorthol et al., 2010).

With respect to Reasons for stopping driving it is important to note that physical incapacity was only the third most cited reason and second for men, further reinforcing the complexity of the process of driving cessation as not just one of health and physical status. The two reasons which produced significantly different proportions in men and women were Reasons not related to health, which was more prevalent among women and Problems with eyesight, which were more prevalent among men. Taken in conjunction with the above noted greater rate of decline in driving among women with age, it would seem that the reasons why women give up driving quicker than men are poorly understood and need more investigation (Dickerson et al., 2017). Three Reasons for ceasing driving were impacted by Age category. Becoming too expensive was cited more frequently than expected by the youngest group and less so by the oldest group. Presumably the youngest group may still have financial dependents and concerns relating to issues such as mortgages while the oldest group are likely to no longer have such concerns. Visual impairment, consistent with previous research, was impacted by Age category with it being less prevalent than expected among the younger group and more prevalent among the oldest group. Being Told by family was higher than expected in the oldest group suggesting that family members become more concerned with age. But the legitimacy of these concerns needs to be questioned given that the same pattern does not emerge for doctors, although it is likely in some cases doctors may not discuss or advise on driving (e.g., Puvanachandra et al., 2008). The fact that the frequency of Physical incapacity being cited as a reason was not impacted by Age category was somewhat surprising but may reflect the fact that subjective appreciation of health related status may not reflect reality (Schneider et al., 2004).

Looking at the impact of Age, Gender and Driving Status on Quality of Life and Loneliness it is clear given the effect sizes that only Driver Status had a meaningful impact on these with being a Current driver conferring an advantage over having ceased driving or never haven driven. Although these are simplistic models it does give an indication of the advantages that are experienced by drivers. Obviously, there are other variables that might be conferring this advantage, and these would need to be teased apart through the use of more sophisticated statistical models.

An interesting area of research relates to exploring the heterogeneity of transport profiles associated with increased heterogeneity and inter-individual variability of later life. One overview of existing studies suggested segmenting older peoples’ transport profiles into four generic segments: (1) an active car-oriented segment; (2) a car-dependent segment, restricted in mobility; (3) a mobile multi-modal segment; (4) and a segment depending on public transport and other services (Haustein and Siren, 2015). The current data did not lend themselves to such an analysis but future research may benefit from ensuring such profiles can be identified should they exist.

## Ethics Statement

This study was carried out in accordance with the recommendations of the Faculty of Health Sciences Research Ethics Committee at Trinity College Dublin with written informed consent from all subjects. All subjects gave written informed consent in accordance with the Declaration of Helsinki. The protocol was approved by the Faculty of Health Sciences Research Ethics Committee at Trinity College Dublin.

## Author Contributions

MG conducted the analysis and wrote the Materials and Methods, Results, and Discussion. DO wrote the Introduction and contributed to the Discussion.

## Conflict of Interest Statement

The authors declare that the research was conducted in the absence of any commercial or financial relationships that could be construed as a potential conflict of interest.

## Footnotes

1 The original question relating to transport options provided participants with 12 options to select from and these were subsequently collapsed to 5, e.g., the three categories Driven as passenger by family, Driven as passenger by friends and Taxi/hackney were collapsed to Driven by someone else.

2 With eight comparisons the new critical alpha level when a Bonferroni conversion is applied becomes 0.00625. The other six analyses were non-significant at the standard alpha level of 0.05.

3 To make the numbers in each age group as comparable as possible the age bands chosen were 50–59, 60–69, and 70+.
